# Antioxidant, cell-protective, and anti-melanogenic activities of leaf extracts from wild bitter melon (*Momordica charantia* Linn. var. *abbreviata* Ser*.*) cultivars

**DOI:** 10.1186/s40529-014-0078-y

**Published:** 2014-12-10

**Authors:** Tsung-Hsien Tsai, Ching-Jang Huang, Wen-Huey Wu, Wen-Cheng Huang, Jong-Ho Chyuan, Po-Jung Tsai

**Affiliations:** 1 Department of Dermatology, Taipei Municipal Wan Fang Hospital and Taipei Medical University, Taipei, Taiwan; 2 Institute of Microbiology and Biochemistry, and Department of Biochemical Science and Technology, National Taiwan University, Taipei, Taiwan; 3 Department of Human Development and Family Studies, National Taiwan Normal University, 162 Hoping E. Rd., Sec. 1, Taipei, 10610 Taiwan; 4 Hualien District Agricultural Research and Extension Station, Hualien, Taiwan

**Keywords:** Wild bitter melon leaf, Antioxidant, Cyto-protection, Anti-melanogensis

## Abstract

**Background:**

Several wild bitter melon (WBM; *Momordica charantia* Linn. var. *abbreviata* Ser.) cultivars were developed in Taiwan. However, little information is available regarding biological function of WBM leaf. Therefore, the objectives of this study were to investigate the nutrient content, antioxidant, cell protection and anti-melanogenic properties of wild bitter melon leaf.

**Results:**

Methanolic leaf extracts were prepared from a variety and two cultivars of WBM. All extracts exerted potent nitric oxide and hydroxyl radical scavenging capacities. Furthermore, all extracts effectively reduce the production of reactive oxygen species and prevent cell death in UVB-irradiated HaCaT keratinocytes. The cell protective effect of leaf extract was also investigated by the prevention of HaCaT cells from sodium nitroprusside or menadione-induced toxicity, and significant cyto-protective activities were observed for all of them. Additionally, all extracts significantly suppressed tyrosinase activity and melanin levels in B16-F10 melanocytes.

**Conclusions:**

WBM leaf extract showed significant antioxidant, cyto-protective and anti-melanogenic activities. These findings suggested that WBM leaves may be beneficial for preventing the photo-oxidative damage and melanogenesis of skin.

**Electronic supplementary material:**

The online version of this article (doi:10.1186/s40529-014-0078-y) contains supplementary material, which is available to authorized users.

## Background

Oxidative stress has been thought to play an important role in the pathogenesis of diseases such as cancer, cardiovascular disease, atherosclerosis, diabetes mellitus, and neurodegenerative disorders (Valko et al. [2007]). Ultraviolet (UV) irradiation is the most well-known environmental skin aggressor. Skin, the largest organ of human body, is a physiological barrier that protects the organism against pathogens and chemical or physical damage. Exposure of UV leads to increased reactive oxygen species (ROS) production, which alters gene and protein structure and function (Masaki [2010]). ROS include free radicals such as superoxide anion (O2^*•−*^), hydroxyl radical (^•^OH), and non-radical molecules like hydrogen peroxide (H_2_O_2_), singlet oxygen (^1^O_2_), nitric oxide (NO), etc. ROS are involved in the pathogenesis of several skin disorders including photosensitivity diseases and some types of cutaneous malignancy (Bickers and Athar [2006]). Additionally, ROS may accelerate aging process and cause uneven pigmentation. UVB radiation has been shown to augment nitric oxide and peroxynitrite formation in keratinocytes (Deliconstmtinos et al. [1996]). NO production may contribute to the regulation of UV-induced pigmentation. NO derived from keratinocytes increases the amount of the melanogenic factor tyrosinase and then induces melanogenesis (Masaki [2010]). Antioxidant agents may therefore play a protective role during the development of ROS-mediated skin disorders.

Wild bitter melon (WBM; *Momordica charantia* L.var. *abbreviata* Seringe) is a variety of bitter melon (*M. charantia*) in Taiwan. WBM fruit is commonly consumed as vegetable and possesses potent antioxidant and free radical scavenging activities (Wu and Ng [2008]). The young shoots and leaves of WBM are traditionally eaten as greens by the Amis, one of the indigenous peoples of Taiwan. The young tender leaves of bitter melon are also eaten as a vegetable in the Philippines and Indonesia. Besides being as a vegetable, the pounded leaves of bitter melons are applied to the body for skin diseases and burns in Malaysia and India (Lim [2012]). In fact, leaf extracts of bitter melon have been demonstrated to have broad-spectrum antimicrobial (Khan and Omoloso [1998]) and potent antioxidant activities (Kubola and Siriamornpun [2008]).

To date, the genetic improvement of WBM has been achieved to improve their agronomic characteristics such as disease resistance, environmental tolerance and fruit quality. Over the years, several WBM cultivars were developed in Taiwan (Lu et al. [2011], [2012]). WBM fruit extract and its components have been shown to possess numerous pharmacological actions including the activation of peroxisome proliferator-activated receptor, antibacterial, anti-inflammatory, and antioxidant activities (Lu et al. [2011], [2012]; Hsu et al. [2012]). But scientific literatures concerning chemical and biological properties of WBM leaves remain limited.

In this study, we intended to analyze the nutrient contents of fresh leaves from a variety and two cultivars of WBM, and to investigate the total phenolic contents and the antioxidant properties of WBM leaf extracts using cell-free and cell-based assays. In addition, the effects of WBM leaf extracts on tyrosinase activity and melanin levels in B16-F10 melanoma cells were determined. Such a study would contribute to the current knowledge relating to the nutrients and biological functions of WBM leaf.

## Methods

### Preparation of extracts

A variety of WBM (WV) and two cultivars of WBM, Hualien-1 (HL-1) and Hualien-2 (HL-2), used in the present study were cultured in the Hualien District Agricultural Research and Extension Station, Hualien, Taiwan. A voucher specimen has been deposited in the Department of Human Development and Family Studies, National Taiwan Normal University. After washing with water, leaves of WBM were air-dried. They were ground by a blender and then extracted with methanol. Briefly, 10 g of fine-ground WBM leaves was extracted with 100 mL of methanol at room temperature for 4 h. After extraction, the mixture was filtered, and the residue was re-extracted with 100 mL of fresh methanol by stirring overnight. The combined methanol solutions were centrifuged at 12,000 × *g* for 10 min and evaporated on a rotary evaporator to get methanolic extracts. The methanolic extracts were reconstituted in dimethyl sulfoxide (DMSO) to a concentration of 400 mg/mL for the subsequent experiments. The yields of WV, HL-1, and HL-2 extracts were 29.1%, 22.3%, and 23.5%, respectively.

### Nutrient content determination of fresh WBM leaves

Moisture, crude fat, crude protein, crude fiber and ash determinations of WBM fresh leaves were conducted following the procedure of the AOAC “Official Methods of Analysis” 14th ed et al. ([1984]). Vitamin C was determined by a colorimetric method of Zhang et al. ([2009]), with modifications. Two-gram dried ground WBM leaf was stirred with 30 mL distilled water for 30 min at room temperature. The homogenization was filtered and then centrifuged at 3000 × *g* for 15 min. The supernatant was collected and diluted with distilled water to total of 50 mL. A 10:1 dilution was made by taking 1.0 mL of this solution and adding distilled water to 10 mL. A 2 mL volume of trichloro-acetic acid (10%) was added to this suspension and placed for 5 min in an ice bath. Afterwards, 2 mL of Folin–Ciocalteu’s phenol reagent was added and vortexed. After 10 min at room temperature, the absorbance was measured at 760 nm against distilled water as a blank. The vitamin C content was estimated through the calibration curve of ascorbic acid.

### Total phenolic content of WBM leaf extract

Total phenolic content of leaf extracts was evaluated using spectrophotometric analysis with Folin-Ciocalteu reagent as described by Tsai et al. ([2008]). Briefly, Folin–Ciocalteu phenol reagent was added to the reconstituted samples and held for 3 min. Then 2 ml of 10% (w/v) sodium carbonate solution were added and allowed to stand at room temperature for 30 min. The absorbance at 765 nm was measured. The total phenolic content was calculated by a standard curve prepared with gallic acid and expressed as milligrams of gallic acid equivalents (GAE) per gram of solid of extract.

### Determination of DPPH radical-scavenging activity of WBM leaf extracts

The 2,2-diphenyl-1-picrylhydrazyl (DPPH•) radical-scavenging capacity of each extract was measured as described earlier (Tsai et al. [2008]). Briefly, 20 μL of each sample or 100% DMSO (as a negative control) were allowed to react with 200 μL of freshly prepared 200 μM DPPH ethanolic solution in a 96-well microplate. The reaction mixture was mixed and left to stand for 10 min. The absorbance at 540 nm was determined against a blank of DMSO. The DPPH radical-scavenging activity of WBM extract was calculated as follows: (1-[A _sample_ –A _blank of sample_/A_DMSO_ –A _blank of DMSO_]) × 100%.

### Determination of NO-scavenging activity of WBM leaf extracts

The NO-scavenging activity of each extract was also measured. Nitric oxide was generated from sodium nitroprusside (SNP) and measured by the Griess reagent (Sumanont et al. [2004]). Briefly, 50 μL of serial diluted sample extract (0.5 ~ 20 mg/mL) was pipetted into a 96-well flat-bottomed plate. Following this, 50 μL of 10 mM sodium nitroprusside dissolved in PBS was added into each well and the plate was then incubated under light at room temperature for 150 min. Finally, an equal volume of Griess reagent was added into each well to measure the nitrite content. The NO-scavenging activity of WBM leaf extract was calculated as follows: (A _DMSO_ – A _sample_)/A _DMSO_ × 100%.

### Determination of superoxide-scavenging activity of WBM leaf extracts

The superoxide scavenging activity of WBM leaf extract was measured using non-enzymatic generation of superoxide anions (Robak and Gryglewski [1988]). Briefly, the reaction mixture contained various concentrations of leaf extracts (0.5-20 mg/mL), 80 μM phenazine methosulphate, 624 μM NADH and 200 μM nitro blue tetrazolium (NBT) in phosphate buffer after 15 min of the incubation at room temperature. The result was measured at 560 nm against blank samples without NADH. The percentage of scavenging effect was expressed as % of scavenging activity =1-[A_560_ sample –A_560_ blank of sample/A_560_ control –A_560_ blank of control] × 100%.

### Determination of hydroxyl radical-scavenging activity of WBM leaf extracts

The scavenging activity of leaf extracts on hydroxyl radicals produced by the Fenton reaction was evaluated with their quenching effects on the chemical luminescence (CL) signal of the Fe(II)–H_2_O_2_–luminol system (Cheng et al. [2003]). Reaction mixtures (200 μL) included luminol (4 μM), Fe^2+^ (4.6 μM)-EDTA (2.3 μM), H_2_O_2_ (24 mM), and tested samples (0.02-1 mg/mL of WBM leaf extract). The reaction was initiated by adding Fe^2+^-EDTA, luminol followed by H_2_O_2_. The chemiluminescent reaction was performed in a KH_2_PO_4_-NaOH buffer (pH 7.5) at room temperature. Luminescence intensity was monitored over wavelengths at 460 nm with Synergy HT multidetection microplate reader (Biotek Instruments, Winooski, VT, USA). The CL peak values were recorded in the absence (I_0_) or presence (Ii) of leaf extracts. The inhibitory rate (IR) was calculated as IR = (1 – I_i_ / I_0_) × 100%.

### UVB-irradiated HaCaT keratinocytes

The immortalized human keratinocyte cell line HaCaT was maintained in Dulbecco’s modified Eagle's medium (DMEM, Gibco, Carlsbad, CA, USA) supplemented with 10% heated-inactivated fetal bovine serum (FBS), penicillin (100 U/mL), and streptomycin (100 μg/mL). These cells were incubated at 37°C in a humidified atmosphere with 5% CO_2_. The probe 2’, 7’-dichlorofluorescein diacetate (H_2_DCF-DA; Sigma, St. Louis, MO, USA) was used to monitor the intracellular ROS generation. HaCaT cells (2 × 10^5^ cells/well) were seeded on 6-well plates for 24 h incubation and then pre-treated with WBM leaf extracts at the indicated concentrations for additional 24-h incubation. After incubation, cells were washed with PBS and then irradiated with 80 mJ/cm^2^ of UVB (Vilber Lourmat, France). In parallel, non-irradiated cells were treated similarly and were kept in the dark in an incubator for the time of UVB treatment. After UVB irradiation, cells were harvested and washed twice with PBS, and then re-suspended in 10 μM H_2_DCF-DA at 37°C for 30 min incubation. Stained cells were washed with PBS and re-suspended in PBS. ROS generation of HaCaT cells was determined by flow cytometry (FACscan, Hercules, CA, USA) using 488 nm for excitation and 525 nm for emission. Mean fluorescence intensity (MFI) detected by FLl channel was analyzed using the Windows Multiple Document Interface software (WinMDI 2.8). Data were expressed as percentages of vehicle control values.

To determine the protective effect of WBM leaf extracts against UVB-induced cytotoxicity, HaCaT cells were seeded in 96-well plates at a density of 2 × 10^4^ cells/well for 24 h incubation and then pre-treated with WBM leaf extracts at the indicated concentrations for additional 24-h incubation. After incubation, cells were washed with PBS and then irradiated with 80 mJ/cm^2^ of UVB. In parallel, non-irradiated cells were treated similarly and were kept in the dark in an incubator for the time of UVB treatment. After challenge of HaCaT cells with UVB, the cells were incubated in fresh DMEM with 10% FBS at 37°C for 30 min or 16 h, and collected for further analysis. The 3-(4, 5-dimethylthiazol-2-yl)-2, 5-diphenyltetrazolium bromide (MTT) assay was performed to determine cellular viability.

### Cell protection effect of WBM leaf extracts against SNP-induced toxicity

The protective effect of WBM leaf extract against cell death induced by sodium nitroprusside (SNP) was determined by the method of Bastianetto et al. ([2010]). SNP is a well-known NO releasing with purported toxic and apoptotic effects in keratinocytes (Bastianetto et al. [2010]). SNP-induced toxicity was performed in HaCaT cells (5 × 10^4^/well) plated in 96 wells. After 24 h, the medium was removed and replaced with medium contain SNP (2 mM) in the presence or absence of leaf extracts (50 ~ 200 μg/mL). Cell viability was determined 24 h later using the MTT assay.

### Cell protection effect of WBM leaf extracts against menadione-induced toxicity

The protective effect of WBM leaf extract against cell death induced by menadione (2-methyl-1, 4-naphthoquinone) was measured by the method of Klausc et al. ([2010]). Menadione is highly cytotoxic, strongly induced ROS formation in human HaCaT keratinocytes. Menadione-induced toxicity was performed in HaCaT cells (5 × 10^4^/well) plated in 96 wells. After 24-h incubation, the medium was removed and replaced with medium contain menadione (50 μM) in the presence or absence of WBM leaf extracts (50 ~ 200 μg/mL). Cell viability was determined 24 h later using the MTT assays.

### Determination of cellular tyrosinase activity in melanoma cells

The established murine B16-F10 melanoma cell line offers a melanogenesis model (Yokozawa and Kim [2007]). The B16-F10 cell line (BCRC 60031) was obtained from the Bioresource Collection and Research Center (Hsinchu, Taiwan) and was cultured in DMEM (Gibco) supplemented with 10% heat-inactivated FBS, penicillin (100 U/mL), and streptomycin (100 μg/mL) at 37°C in a humidified atmosphere with 5% CO_2_. The effect of WBM leaf extracts on cell viability of B16-F10 melanoma cells was first investigated. B16-F10 melanoma cells (1 × 10^4^ cells/well) were seeded in 96-well culture plates for 24 h prior to use. The cells were treated with various concentrations of 50 ~ 250 μg/mL of WBM leaf extracts for 24 h. The cell viability was evaluated using the MTT assays.

The tyrosinase activity in B16-F10 cells was examined by measuring the rate of oxidation of L-DOPA (Yokozawa and Kim [2007]). B16-F10 cells (2.5 × 10^4^ cells/well) were plated in 24-well dishes for 24 h incubation before the use. The cells were then incubated in the presence or absence of 25 ng/mL α-melanocyte stimulating hormone (α-MSH) and various concentrations of WBM leaf extracts for 48 h. In addition, kojic acid [5-hydroxy-2-(hydroxymethyl)-4-pyrone], a popular inhibitor of tyrosinase (Kahn [1995]) was used as a positive control. The cells were lysed in 200 μL of 50 mM sodium phosphate buffer (pH 6.8) containing 1% Triton X-100 and 0.1 mM phenylmethylsulfonyl fluoride and then frozen at −80°C for 30 min. After thawing and mixing, cellular extracts were clarified by centrifugation at 12,000 × g for 30 min at 4°C. The supernatant (80 μL) and 20 μL of L-DOPA (2 mg/mL) were placed in a 96-well plate, and the absorbance at 492 nm was read for 30 min at 37°C using a micro-plate reader.

### Determination of melanin content in melanoma cells

Melanin content was measured as described previously (Aoki et al. [2007]) with modifications. B16-F10 cells were incubated in the presence or absence of 25 ng/mL α-MSH and various concentrations (50 ~ 200 μg/mL) of leaf extracts for 48 h. The cells were rendered soluble in 1 N NaOH containing 10% DMSO at 60°C for 30 min, and then 200 μL portions of crude cell lysate was transferred into 96-well plates. Melanin concentration was calculated by the absorbance measured at 405 nm through the calibration curve of melanin.

### Statistical analysis

All data are presented as the mean ± standard deviation (SD). Statistical analyses were performed using Statistical Package of Social Science version 17.0 for Windows (SPSS Inc., Chicago, Illinois, USA). Analysis of variance was performed by ANOVA procedures. Significant differences between means between groups were determined using Duncan’s multiple range tests at a level of *p* < 0.05.

## Results

### Nutrient content of WBM leaves

Moisture, crude protein, crude fat, crude fiber, and ash content (%) of WBM leaves were reported on dry-weight basis and given in Table 1. In fresh WBM leaves, crude fat, crude protein, and crude fiber contents ranged from 0.74% to 1.22%, 4.22% to 6.26%, and 1.59% to 1.92% on dry-weight basis, respectively. Ash contents in WBM leaves ranged from 2.5% to 3.76% on dry-weight basis. Vitamin C contents in WBM leaves ranged from 1647.3 to 2059.0 μg/g dry basis.

**Table 1:** **Mositure, crude protein, crude fat, crude fiber, and ash content and vitamin C of fresh wild bitter melon leaves (on dry-weight basis)**

Variety and cultivars	Moisture (%)	Crude protein (%)	Crude fat (%)	Crude fiber (%)	Ash (%)	Vitamin C (μg/g dry basis)
WV	83.20	5.28	0.99	1.90	3.76	1647.32
HL-1	81.22	6.26	1.22	1.92	2.85	1920.14
HL-2	83.77	4.22	0.74	1.59	2.50	2059.03

### Total phenolic content of WBM leaf extract

The total phenolic content of WBM leaf extracts was determined as gallic acid equivalents (Table 2). Among three extracts tested, WV had the highest phenolic content (34 mg GAE/g extract), followed by HL-1 and HL-2 (26 mg GAE/g extract).

**Table 2:** **Total phenolics content and radical-scavenging capacity of methanolic extracts from wild bitter melon leaves**

Variety and cultivars	Total phenolics (mg GAE/g)	IC_50_values (mg/mL)			
		DPPH	NO	Superoxide	Hydroxyl radical
WV	33.70 ± 0.48^b^	4.79 ± 0.30^a^	0.79 ± 0.01	5.77 ± 0.10^a^	0.026 ± 0.003^a^
HL-1	25.86 ± 0.36^a^	28.00 ± 0.83^c^	0.76 ± 0.02	9.12 ± 0.22^c^	0.042 ± 0.004^b^
HL-2	25.94 ± 0.35^a^	20.39 ± 1.12^b^	0.80 ± 0.02	7.71 ± 0.35^b^	0.022 ± 0.004^a^

GAE: Gallic acid equivalent. Data are expressed as the mean ± SD. Values in a column followed by the same superscript letter are not significantly different as determined by Duncan's multiple tests.

### Free radical-scavenging activity of WBM extracts

**Figure 1:**
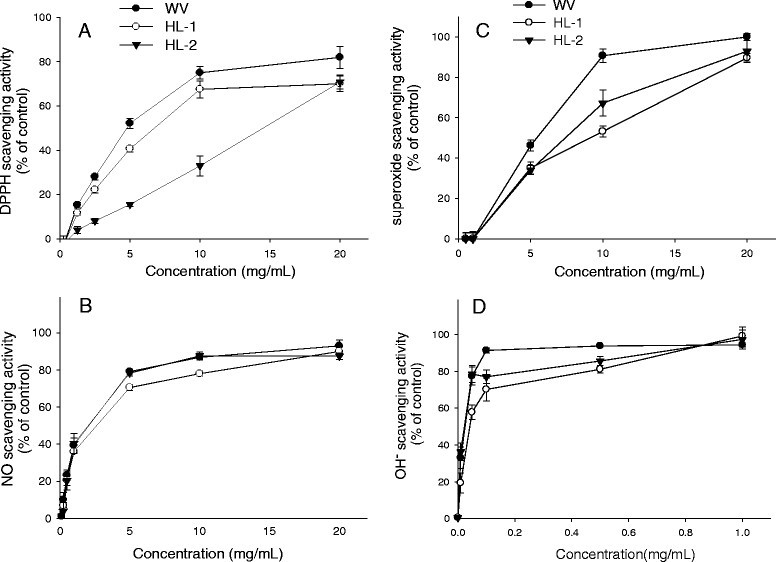
**Scavenging activities of WBM leaf extracts against the DPPH (A), nitric oxide (B), superoxide anion (C), and hydroxyl radical (D).** Data are given as the mean ± SD (n = 3). Radical-scavenging capacity of leaf extract was represented as % of vehicle control.

The free radical-scavenging properties of leaf extracts were examined (Figure 1) and then expressed as the IC_50_ which is the concentration of leaf extract that causes 50% inhibition of respective radical generation (Table 2). The DPPH radical scavenging assay is a simple and widely used screening for bioactive compound discovery. The scavenging activity of WBM leaf extracts on DPPH radicals is in a concentration-dependent manner (Figure 1A). The IC_50_ values of WV, HL-1, and HL-2 extracts on DPPH scavenging activity were 4.79, 28.0, and 20.39 mg/mL, respectively (Table 2). Among tested leaf extracts, WV is the strongest DPPH radicals’ scavenger. HL-1 extract is the least potent scavenger of DPPH.

The NO-scavenging activity of leaf extracts were measured to have IC_50_ values of 0.79, 0.76, and 0.80 mg/mL for WV, HL-1, and HL-2 extracts, respectively (Figure 1B, Table 2), where there was no statistically significant difference observed among the variety and cultivars.

WBM leaf extracts also caused a concentration-dependent inhibition of superoxide anion (Figure 1C). The respective IC_50_ values of WV, HL-1, and HL-2 extracts on superoxide scavenging activity were 5.77, 9.12, and 7.71 mg/mL (Table 2), indicating that WV extract is more potent scavenger of superoxide anion than others.

The hydroxyl radical-scavenging capacity of leaf extracts is shown in Figure 1D. The respective IC_50_ values of WV, HL-1, and HL-2 extracts were 26, 42, and 22 μg/mL (Table 2). WV and HL-2 had more effective scavenging activity than HL-1.

### WBM leaf extracts inhibit UV-induced ROS production and keratinocyte death

**Figure 2:**
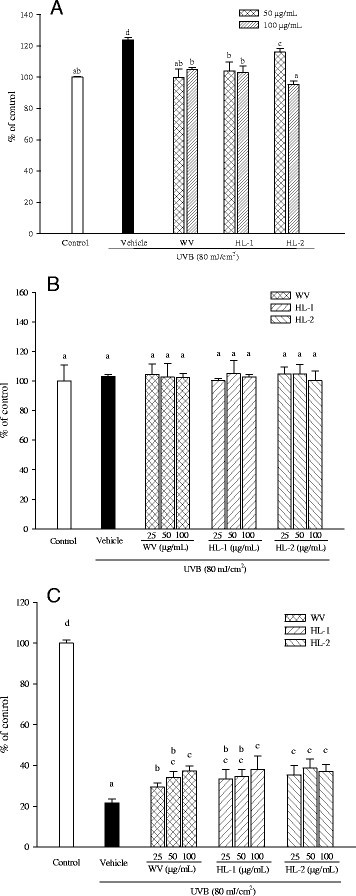
**Effects of WBM leaf extracts on UV-induced ROS production and cytotoxicity in HaCaT keratinocytes.** After exposure of UVB (80 mJ/cm^2^), the cells were further incubated at 37°C for 30 min and then ROS generation was determined by flow cytometry. ROS production was represented as % of control without UVB irradiation. **(A)** After exposure of UVB (80 mJ/cm^2^), the cells were further incubated at 37°C for 30 min **(B)** or 16 h **(C)** and then cell viability was measured using the MTT assay. Non-irradiated HaCaT keratinocytes were used as the control. Cell viability is expressed as the percentage of control. Data are presented as the mean ± SD of triplicate determinations. Values with the same letter are not significantly different as determined by Duncan’s multiple range tests.

As shown in Figure 2A, UVB exposure of HaCaT keratinocytes resulted in an immediate and significant elevation of intracellular ROS. Treatment of WBM leaf extracts (50 and 100 μg/mL) prior to UVB irradiation significantly inhibited intracellular ROS generation. All leaf extracts effectively inhibited UVB-induced ROS production. After UVB exposure, HaCaT cells were further incubated at 37°C for 30 min (Figure 2B) or 16 h (Figure 2C). The results showed that UVB irradiation did not significantly affect cell viability for further 30 min incubation. All three WBM leaf extracts did not significantly modulate cell viability of HaCaT cells during 30 min incubation (Figure 2B). WBM leaf extract-pretreated HaCaT cells were exposed to UVB and incubated for an additional 16 h. MTT assay showed that cell viability of HaCaT cells was significantly decreased after UVB exposure (Figure 2C). Notably, all three WBM leaf extracts at concentrations of 25, 50, and 100 μg/mL revealed a protective effect on the viability of irradiated HaCaT cells (Figure 2C).

### Cellular protection effect of WBM leaf extracts against oxidants

**Figure 3:**
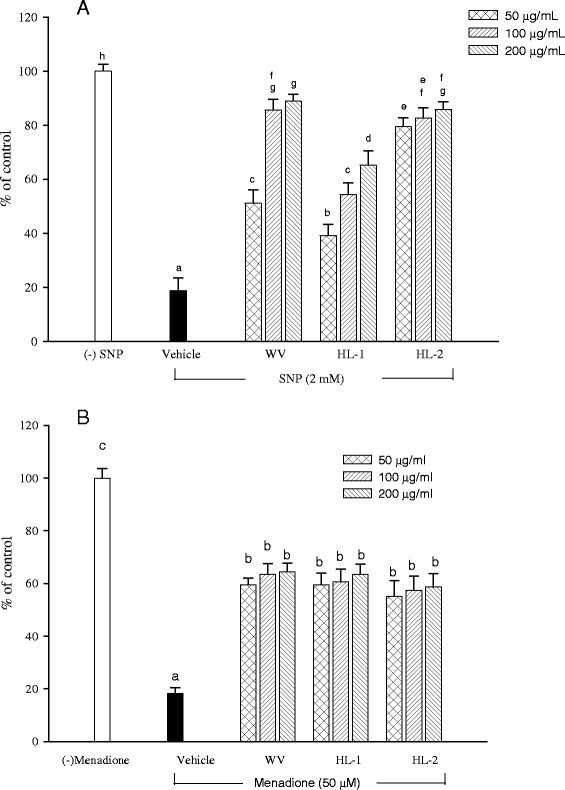
**Effects of WBM leaf extracts against keratinocyte death induced by SNP (A) and menadione (B) treatment.** Cells were exposed to either SNP (2 mM) or menadione (50 μM) in the absence (vehicle) or presence of leaf extracts (50–200 μg/mL). Cell viability was determined 24 hours later using MTT assay. Data are presented as the mean ± SD of triplicate determinations. Values with the same letter are not significantly different as determined by Duncan’s multiple range tests.

Prior to the determination of cellular protection effect, the cytotoxic effect of WBM leaf extracts on HaCaT keratinocytes were examined. HaCaT cells were incubated with WBM leaf extracts at various concentrations for 24-h incubation. The cell viability was determined by MTT methods. All three WBM leaf extracts (up to 300 μg/mL) had no cytotoxic effect on HaCaT cells (data not shown). On the other hand, treatment of HaCaT cells with SNP (2 mM) (Figure 3A) and menadione (50 μM) (Figure 3B) strongly impaired cell viability. To determine the cyto-protection activities, HaCaT keratinocytes were simultaneously exposed to cytotoxic agents either SNP or menadione as well as WBM leaf extracts at various concentrations (Figure 3). All three WBM leaf extracts strongly attenuated SNP-induced toxicity at a concentration of 50 μg/mL (Figure 3A). The HL-2 extract was the most effective one in protecting cells against SNP-induced toxicity with an EC_50_ (effective concentrations) of 31.31 μg/mL, followed by WV (EC_50_ = 49.35 μg/mL), and HL-1 (EC_50_ = 84.72 μg/mL).

We then performed a cell viability assay to evaluate the capacity of WBM leaf extracts to protect HaCaT cells against the toxicity induced by the ROS releasing menadione (Figure 3B). Exposure of HaCaT cells to menadione resulted in cell death which was reduced by WV (EC_50_ = 42.05 μg/mL), HL-1 (EC_50_ = 42.07 μg/mL), and HL-2 (EC_50_ = 43.55 μg/mL). According to EC_50_ values, the three varieties were comparable in their cyto-protective effect against menadione-induced oxidative stress.

### Anti-tyrosinase and anti-melanogenic properties of WBM leaf extracts

**Figure 4:**
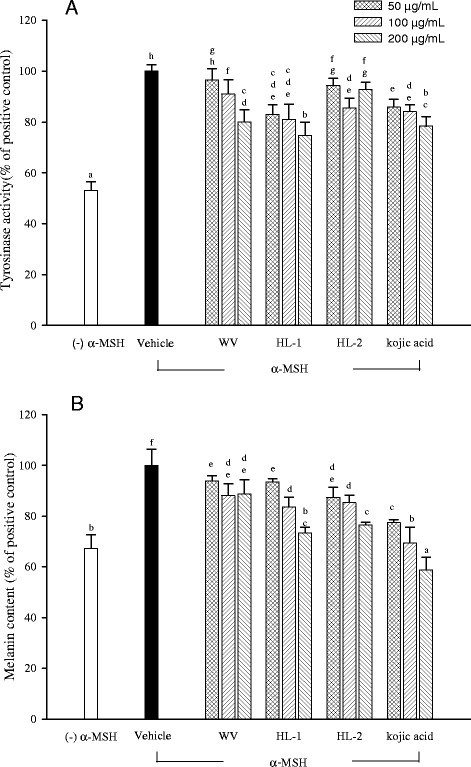
**Effects of WBM leaf extract on cellular tyrosinase activity (A) and melanin level (B) of α-MSH treated B16-F10 melanocytes.** Each value is expressed as the mean ± SD (n = 3). Values with the same letter are not significantly different as determined by Duncan’s multiple range tests.

To investigate the anti-melanogenic activity in cellular system, the cytotoxicity of WBM leaf extracts on B16-F10 cells was first evaluated after incubated with extracts for 24 hr. Our data showed that these three extracts (up to 200 μg/mL) did not show any cytotoxic effect on B16-F10 cells (data not shown). Thus, WBM leaf extract (50–200 μg/mL) was used to examine their anti-tyrosinase activity and intracellular melanin formation. As shown in Figure 4, all three WBM leaf extracts exhibited significantly inhibitory effect on tyrosinase activity (Figure 4A). However, at a concentration of 50 μg/mL, the anti-tyrosinase effect of WV was not significant. At a concentration of 200 μg/mL, HL-1 extract exhibited more significant anti-tyrosinase activity than others. The inhibitory effect of HL-1 extract (ranged from 50 to 200 μg/mL) on tyrosinase activity were similar to kojic acid, a well-known tyrosinase inhibitor (Figure 4A).

All three WBM leaf extracts significantly suppressed melanin formation in B16-F10 cells (Figure 4B). At a concentration of 200 μg/mL, WV, HL-1, and HL-2 extracts and kojic acid reduced melanin production of 11.29%, 26.63%, 23.44% and 41.2%, respectively. The anti-melanogenis effect of HL-1 is higher than those of WV and HL-2, but lower than that of kojic acid.

## Discussion

In this study, the cultivars of wild bitter melon leaves are proved in possessing antioxidant and anti-melanogenic properties. Although there are some differences in the degree they exerted, however, all of them possess antioxidant to reduce UVB-induced ROS generation and prevent cellular death against oxidants in keratinocytes.

Leafy vegetables are good sources of not only minerals but also vitamins, antioxidants and pigments. The tender leaf tips and leaves of bitter melon are a rich source of minerals, vitamin C, folic acid and vitamin A (Zhang et al. [2009]; Lim [2012]). This study analyzed nutrient content of WBM leaf and provided further information on nutrient contents on WBM leaf for nutritionists and the general public. During the past few decades, the uses of natural antioxidants and plant extracts for human health have received increasing attention.

The findings presented here showed that WBM leaf extracts exhibited antioxidant properties because of their capacity to scavenge various free radicals and to reduce oxidant-induced cellular death. As Figure 1 and Table 2 shown, WBM leaf extracts exerted hydroxyl radical and NO scavenging activities. The most impressive radical-scavenging activity is against hydroxyl radical, which is considered to be the most reactive one among ROS. The IC_50_ values for hydroxyl radial ranged from 22 to 42 μg/mL. The wild variety and HL-2 were superior to HL-1. Leaf extract of Thai bitter melon possesses hydroxyl-radical scavenging activity with an IC_50_ value of 167 ± 0.96 mg/mL (Kubola and Siriamornpun [2008]). Lu et al. ([2012]) demonstrated fruit extract of the most effective WBM cultivar in Taiwan exerted potent hydroxyl-radical scavenging activity (IC_50_, 37 μg/mL). These results further supported that leaf and fruit of WBM possess strong hydroxyl-radical scavenging activity.

Numerous phytochemicals such as momordicine, kuguacins, and phenolics, have been isolated from bitter melon leaves (Lim [2012]; Kubola and Siriamornpun [2008]). The new finding triterpenoids isolated from the stems and fruits of bitter melons show their antioxidant property (Liu et al. [2010]; Lin et al. [2011]). Kubola and Siriamornpun ([2008]) reported that leaf extract of bitter melon possess antioxidant activity, based on DPPH radical-scavenging activity and ferric acid reducing power. The predominant phenolic compounds in the leaf of bitter melon are gallic acid, followed by caffeic acid and catechin (Kubola and Siriamornpun [2008]). We recently investigated phenolic compounds of these three WBM leaf extracts using HPLC methods (Kubola and Siriamornpun [2008]; Zhang et al. [2009]) with some modifications. The phenolic compounds found in WBM leaves were gallic acid, salicylic acid, cinnamic acid, myricetin, quercetin, and luteolin (Additional file 1). Because of the diversity and complexity of the natural mixtures of antioxidants in WBM leaf extracts, it is rather difficult to characterize all of compounds by HPLC. Further work is still required to verify the anti-oxidative constituents of WBM leaves.

Skin is constantly exposing to environmental insults, among all, UV light is thought to be the most harmful one. UV exposure can cause oxidative stress and inflammation. Eating plenty of green leafy vegetables has been considered to be beneficial for photoprotection (Mukhtar [2003]). Phytonutrients with antioxidant activity have been considered to be beneficial for attenuating UV-caused oxidative stress and oxidative stress-mediated skin disorders (Evans and Johnson [2010]). To elucidate the antioxidant activity exerted by WBM leaf extracts, the intracellular ROS generation in UV-irradiated keratinocyte was carried out using an oxidant-sensitive fluorescent probe DCF-DA. By measuring the intercellular ROS scavenging activity of WBM leaf extracts, the results has shown that pre-treatment of extracts could reduce the intracellular ROS production induced by UV within cells. Moreover, all three WBM leaf extracts showed the protective effect on the viability of UVB-irradiated keratinocytes (Figure 2). Therefore, WBM leaf extract could be proposed as a photo-protective agent preventing harmful effects of UVB exposure on human keratinocytes.

As WBM leaf extracts showed antioxidant effects against free radicals and intercellular ROS, their cell protective effects on keratinocytes against the cellular damage induced by SNP (a NO donor) and menadione (a ROS donor) were evaluated (Figure 3). The cell protective property of WBM leaf extracts against SNP or menadione-induced cell death can be observed herein. Kumar et al. ([2010]) reported that fruit extract of bitter melon showed significant cytoprotection against oxidants on fibroblasts and keratinocytes. In this study, WBM leaf extract protect HaCaT cells from damage caused by SNP, which implies that WBM leaf extracts are able to block the harmful events induced by NO overproduction, a process relevant to premature skin aging occurring upon long term UV exposure (Weller [2003]). Similarity, all three WBM leaf extracts possessed cyto-protection against menadione-induced damage on HaCaT keratinocytes, suggesting its ability to reduce superoxide-mediated stress in skin. In considering that ROS plays an important role in the pathogenesis of many skin disorders and chronologic skin aging (Bickers and Athar [2006]), these findings presented in this study suggest that WBM leaf is a good source of natural antioxidants and may prevent the ROS-mediated skin disorders.

Clinical changes recognizable as photoaging include wrinkle, inelasticity, telangiectasia and pigmentary change. Although not detrimental in nature, hyperpigmentation can be a big cosmetic concern, especially among Asian. The inhibition of melanogenesis is a critical target for skin-whitening cosmetic and treatment of abnormal pigmentation (Yokozawa and Kim [2007]). Since tyrosinase is a rate-limiting enzyme of melanogenesis, inhibition on its action is one major strategy to treat hyperpigmentation. The anti-tyrosinase and anti-melanogenic activity of natural substances have been studied extensively. Fruit extracts of bitter melon have been shown to exert significant inhibitory effect on mushroom tyrosinase (Kamkaen et al. [2007]; Masuda et al. [2007]; Baurin et al. [2002]). Kamkaen et al. ([2007]) reported that methanolic extract of bitter melon fruit can exert a significant mushroom tyrosinase inhibition (78.9% compared to positive control of kojic acid). Masuda et al. ([2007]) reported that ethanol extract of bitter melon fruit can show 21.7% of mushroom tyrosinase inhibitory activity at a concentration of 0.5 mg/mL. Similar finding was reported in previous study that used propylene glycol/deionized water extract of bitter melon plants with 32% inhibition of mushroom tyrosinase (Baurin et al. [2002]). In our preliminary study, WBM leaf extracts exhibited suppressive effect on mushroom tyrosinase activity at a concentration of 5 mg/mL. It is not yet known whether WBM leaf extract has anti-melanogenic activity in cellular assay. In the experiments reported here, HL-1 showed more potent anti-tyrosinase and anti-melanogenic activities than others. Regarding the inhibitory effect on tyrosinase activity, HL-1 extract was similar to kojic acid at each tested concentration (Figure 4). Kojic acid shows its anti-tyrosinase activity by chelating the copper ion in the active site of tyrosinase (Kahn [1995]). However, the inhibitory mechanism or effective constituents of WBM leaf extract is still unclear. Kim et al. ([2008]), Yokozawa and Kim ([2007]), and Kim ([2007]) reported that the antimelanogenic action of some agents is related to their antioxidant activity. Concerning the action mechanism of anti-tyrosinase agents, blocking of oxidative pathway (Kim [2007]) and binding of enzyme activity sites (Curto et al. [1999]) may be involved in inhibiting the catalytic reaction of tyrosinase. Previous studies demonstrated that gallate and its derivatives (Kim [2007]; Tanford [1980]) and p-alkoxybenzoic acid derivatives (Chen et al. [2005]) can act as tyrosinase inhibitors. The bulky hydrophobic portions of gallate derivatives were considered to interact with tyrosinase’s hydrophobic protein bulk surrounding the binuclear copper active site and thus gallate derivatives exerted the anti-tyrosinase effect (Tanford [1980]). Since gallic acid was found in WBM leaf extracts, it speculated that gallic acid may contribute, at least partially, to anti-melanogenic activity of WBM leaf extracts. Other unknown active constituents present in WBM leaf extract could also play critical roles in the biological effects. The identification of these functional or bioactive ingredients in WBM leaf extract is an interesting topic for future study.

## Conclusions

In summary, all leaf extracts form WBM variety and cultivars showed antioxidant, cell protection, and anti-melanogenic activities. Among them, HL-1 showed the most potent anti-melanogenic activity. These findings may be used to develop health foods or cosmetics, and to increase the range of applications of traditional agricultural vegetables.

## Additional file

## Electronic supplementary material

Additional file 1:**HPLC profiles of WBM leaf extracts (A) and reference authentic standards (B) detected at 280 nm.** Peaks: 1, gallic acid; 2, salicylic acid; 3, caffeic acid; 4, ferulic acid; 5, cinnamic acid; 6, myricetin; 7, quercetin; 8, luteolin. Chromatographic separations were performed on a C-18 reversed-phase silica Bondclone column (300 × 3.9 mm i.d., 10 μm, Phenomenex, Torrance, CA, USA). The mobile phase was a mixture of solvent A (water/methanol, 98:2), and solvent B (methanol/acetic acid, 98:2) according to a linear gradient elution from 10% B to 80% B during 30 min, at a flow-rate of 1 mL/min. The following gradient was used 10-40% B in 15 min; 40-80% B in 15 min. (DOCX 85 KB)

Below are the links to the authors’ original submitted files for images.

Authors’ original file for figure 1

Authors’ original file for figure 2

Authors’ original file for figure 3

Authors’ original file for figure 4

Authors’ original file for figure 5

Authors’ original file for figure 6
