# Supplementary material for: Antioxidant, cell-protective, and anti-melanogenic activities of leaf extracts from wild bitter melon (Momordica charantia Linn. var. abbreviata Ser.) cultivars
Source: Bot Stud. 2014 Dec 10;55:78. doi: 10.1186/s40529-014-0078-y (PMC5432827; doi:10.1186/s40529-014-0078-y)
Supplement: Supplementary file 1 — Additional file 1:HPLC profiles of WBM leaf extracts (A) and reference authentic standards (B) detected at 280 nm. Peaks: 1, gallic acid; 2, salicylic acid; 3, caffeic acid; 4, ferulic acid; 5, cinnamic acid; 6, myricetin; 7, quercetin; 8, luteolin. Chromatographic separations were performed on a C-18 reversed-phase silica Bondclone column (300 × 3.9 mm i.d., 10 μm, Phenomenex, Torrance, CA, USA). The mobile phase was a mixture of solvent A (water/methanol, 98:2), and solvent B (methanol/acetic acid, 98:2) according to a linear gradient elution from 10% B to 80% B during 30 min, at a flow-rate of 1 mL/min. The following gradient was used 10-40% B in 15 min; 40-80% B in 15 min. (DOCX 85 KB) [file 40529_2014_9078_MOESM1_ESM.docx]

Supplementary Fig. 1.


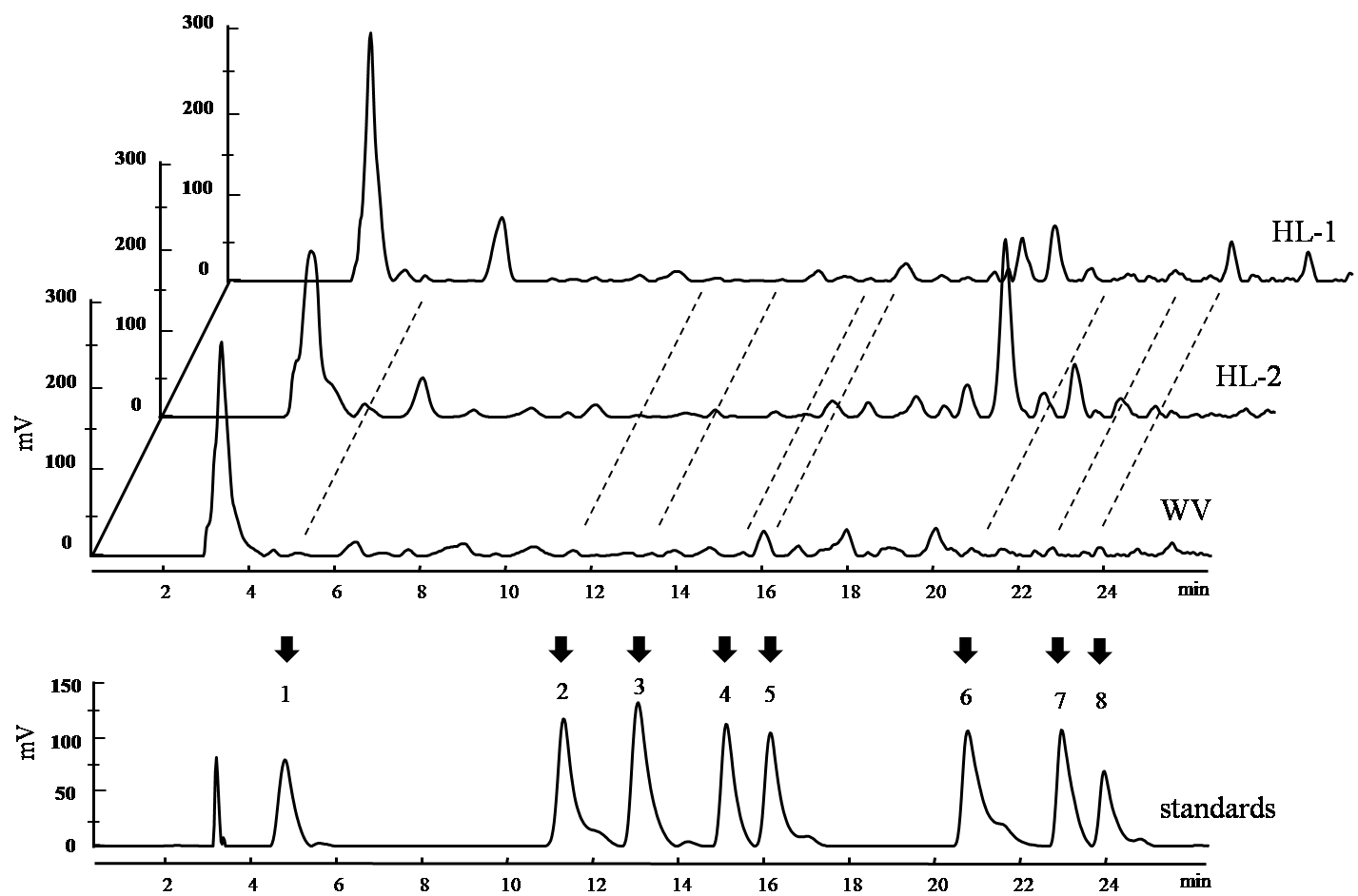


(A)

(B)

Supplementary figure 1. HPLC profiles of WBM leaf extracts (A) and reference authentic standards (B) detected at 280 nm. Peaks: 1, gallic acid; 2, salicylic acid; 3, caffeic acid; 4, ferulic acid; 5, cinnamic acid; 6, myricetin; 7, quercetin; 8, luteolin. Chromatographic separations were performed on a C-18 reversed-phase silica Bondclone column (300×3.9 mm i.d., 10 μm, Phenomenex, Torrance, CA, USA). The mobile phase was a mixture of solvent A (water/methanol, 98:2), and solvent B (methanol/acetic acid, 98:2) according to a linear gradient elution from 10% B to 80% B during 30 min, at a flow-rate of 1 mL/min. The following gradient was used 10-40% B in 15 min; 40-80% B in 15 min.
