# Supplementary figures and images for: Antioxidant, cell-protective, and anti-melanogenic activities of leaf extracts from wild bitter melon (Momordica charantia Linn. var. abbreviata Ser.) cultivars
Source: Bot Stud. 2014 Dec 10;55:78. doi: 10.1186/s40529-014-0078-y (PMC5432827; doi:10.1186/s40529-014-0078-y)

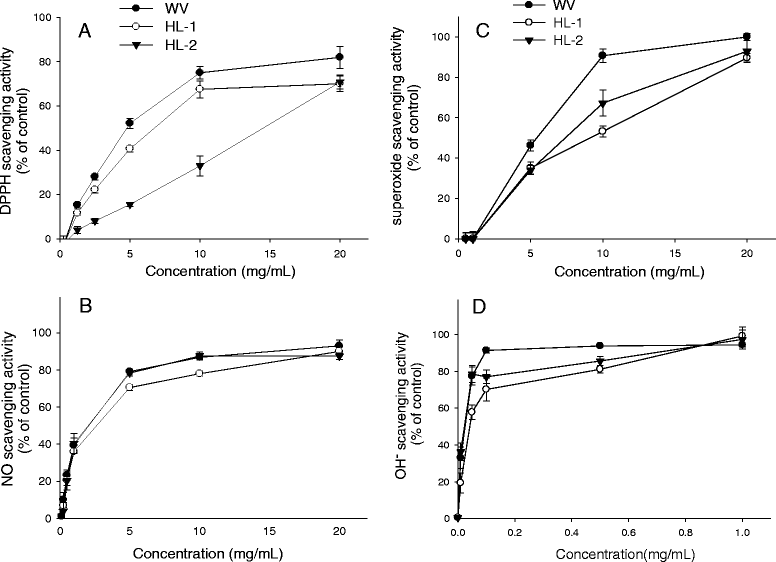

Supplement: Supplementary file 2 — Authors’ original file for figure 1 [file 40529_2014_9078_MOESM2_ESM.gif]

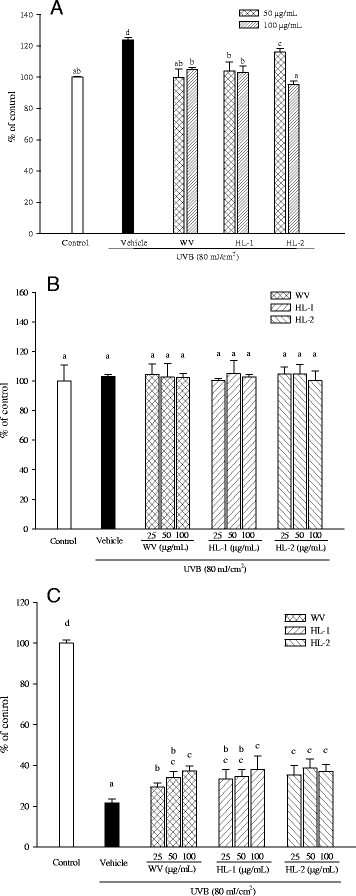

Supplement: Supplementary file 3 — Authors’ original file for figure 2 [file 40529_2014_9078_MOESM3_ESM.gif]

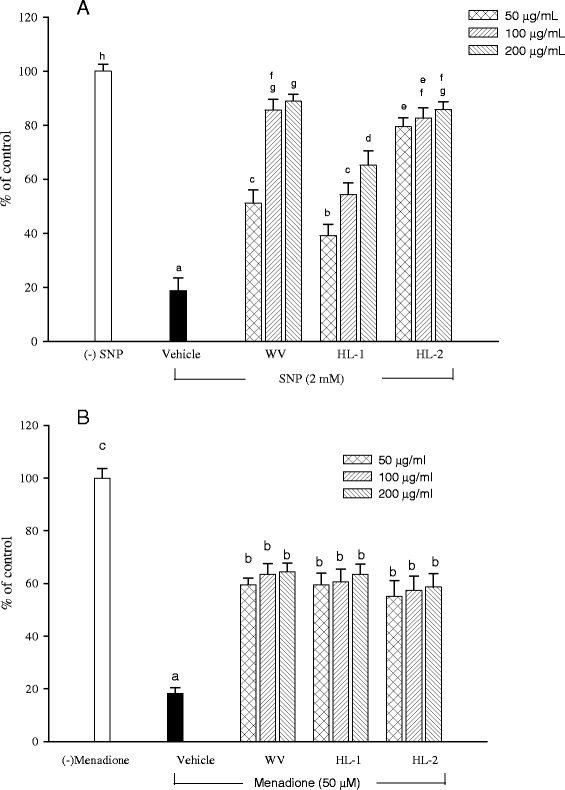

Supplement: Supplementary file 4 — Authors’ original file for figure 3 [file 40529_2014_9078_MOESM4_ESM.gif]

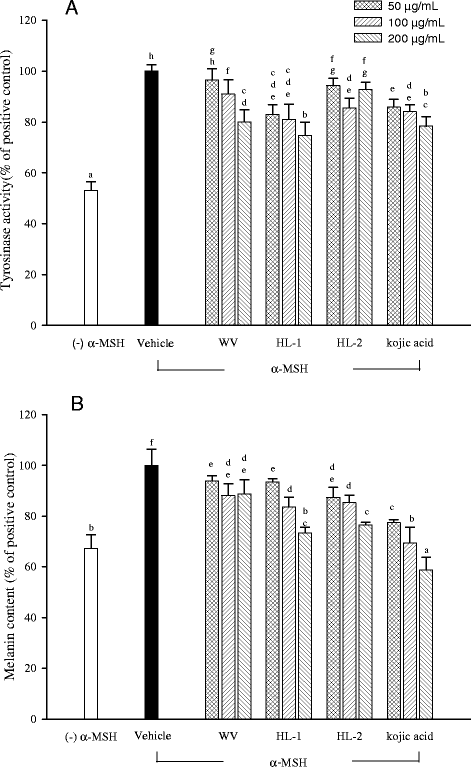

Supplement: Supplementary file 5 — Authors’ original file for figure 4 [file 40529_2014_9078_MOESM5_ESM.gif]
