# Supplementary material for: Antioxidant, cell-protective, and anti-melanogenic activities of leaf extracts from wild bitter melon (Momordica charantia Linn. var. abbreviata Ser.) cultivars
Source: Bot Stud. 2014 Dec 10;55:78. doi: 10.1186/s40529-014-0078-y (PMC5432827; doi:10.1186/s40529-014-0078-y)
Supplement: Supplementary file 7 — Authors’ original file for figure 6 [file 40529_2014_9078_MOESM7_ESM.docx]

Supplementary figure 2.

Supplementary figure 2. HPLC profiles of phenolic standards and WBM leaf extracts detected at 280 nm. Peaks: 1, ascorbic acid; 2, gallic acid; 3, salicylic acid; 4, caffeic acid; 5, ferulic acid; 6, cinnamic acid; 7, myricetin; 8, quercetin; 9, luteolin.
